# Varicella-Zoster Virus Myocarditis: Early Clinical Diagnosis and Outcome

**DOI:** 10.7759/cureus.38015

**Published:** 2023-04-23

**Authors:** Akhila Sai Sree Cherukuri, Naod F Belay, Duaa S Nasereldin, Doaa O Mohammed, Suzan Mohamed, Abuzar Elkhazeen, Naglaa G Ghobriel, Lina Alatta, Wail Alsafi, Yaseen Abdalla, Gamila Brry, Nadir Abdelrahman

**Affiliations:** 1 Geriatrics, Michigan State University, East Lansing, USA; 2 Family Medicine - Geriatrics, Michigan State University College of Human Medicine, East Lansing, USA

**Keywords:** myocarditis, zoster, varicella-zoster virus, vzv vaccine, chickenpox

## Abstract

Varicella-zoster virus (VZV) is a viral infection that causes chickenpox and shingles. Although it is usually self-limiting, it can lead to severe complications, especially in pediatric and immunocompromised patients. VZV was first discovered as a cause of myocarditis in 1953. In this review article, we aim to investigate the early clinical diagnosis of myocarditis in VZV infections and the efficacy of the VZV vaccine in preventing myocarditis. The literature search was done using PubMed, Google Scholar, and Sci-Hub databases. A high VZV mortality rate was noted among adults, infants, and immunocompromised patients. The early diagnosis and treatment of VZV myocarditis can reduce mortality.

## Introduction and background

Varicella, caused by the varicella-zoster virus (VZV), is a common viral illness that mainly affects children, with the highest incidence occurring in those aged one to four years old [1]. Primary viremia develops four to six days after contamination and, similar to other herpes viruses, can unfold to the liver, spleen, and sensory ganglia. After exposure, there is a 14-16-day incubation period. After the primary infection, the virus remains latent, especially in the sensory ganglia, and causes herpes zoster (shingles), which presents with a rash and blisters in a dermatomal distribution associated with pain, itching, and tingling [1]. The start of a rash could be preceded by a modest prodrome. Adults may have fever and malaise for one to two days before the rash appears, but the rash is often the first sign of illness in children. An itchy rash progresses throughout the body rapidly in unvaccinated people [1].

Although reinfections with varicella are thought to be rare [2], a study by Hall et al. [3] suggested that they may occur more commonly than previously recognized, especially in individuals with young age, mild initial infections, and genetic factors that affect the immune response. Early varicella infections, particularly during the first year of life, may not produce an adequate memory cell response, which may predispose a person to either a second infection or reactivation of VZV as herpes zoster [3].

Acute varicella infection is diagnosed with serological tests and polymerase chain reaction (PCR). Real-time PCR test methods are widely used and are the most sensitive and specific available tests. Commercial enzyme-linked immunosorbent assays (ELISA) are recommended for screening. The latex agglutination method is another method to test for serology (IgG) [1]. VZV infection can lead to a wide range of complications, which can be particularly severe and potentially life-threatening for immunocompromised individuals or the elderly [4]. These complications can include bacterial skin infections, pneumonia, encephalitis, herpes zoster ophthalmicus, and myocarditis. In addition, VZV reactivation, known as herpes zoster or shingles, can cause post-herpetic neuralgia, a painful and long-lasting condition that can occur in those who have had chickenpox in the past [4].

VZV infection is a rare cause of cardiovascular complications such as pericarditis, myocarditis, and endocarditis [4]. The first reported case of VZV-induced myocarditis was documented in 1953 [5,6], and since then, this rare but serious complication has been reported more frequently in children and young adults [7]. The pathogenesis of these complications can be attributed to direct cytopathic effects as well as secondary autoimmune reactions leading to the destruction of myocardial cells and subsequent ventricular dysfunction [5,8]. Although the prevalence of VZV antibodies is reported to be around 95% in individuals above the age of 30, there have been sporadic cases of unusual viral presentations, with recent studies in the United States and the United Kingdom suggesting a shift in the age distribution of VZV cases [6,8]. Symptoms can range from a very mild presentation of symptomatic electrocardiographic (EKG) changes and elevated cardiac markers to chest pain, tachycardia, arrhythmias, hypotension, and congestive heart failure [9]. Clinical suspicion supported by serology is sufficient for diagnosing VZV myocarditis; cardiac biopsy, the gold-standard diagnostic test, is not usually performed in clinical practice [10].

Full recovery from the initial VZV infection results in permanent immunity, which is the basis for the current vaccination policy [11]. In 1995, the development of a varicella vaccine led to a significant decrease in both cases and complications [12]. Varicella vaccine is recommended for all susceptible people 12 months of age or older, and disease history is considered a reliable indicator of immunity. In the United States, two live, attenuated VZV-containing vaccinations are approved for use in preventing varicella. The VAR (Varivax) vaccine is a single-antigen chickenpox vaccine, and the MMRV (ProQuad) vaccine is a combined measles, mumps, rubella, and chickenpox vaccine [12]. Children between the ages of 12 and 15 months and four to six years should receive two doses of the chickenpox vaccine, with the efficacy of the first dose efficacy being 82% and the second dose being 92% [12]. The effectiveness of the varicella vaccine has been studied over several years. The vaccine’s efficacy increased from 86.7% in 1997 to 95.7% in 1999 due to residual boosting from natural disease. However, there was a decline in efficacy from 1999 to 2001, which became statistically significant in 2002 when the efficacy declined to 58.4% [13]. Those who received the vaccination experienced fewer fevers than those who are not protected [1,12]. Vaccination against VZV has led to a significant reduction of 70% to 90% and 95% in infections and sequelae, respectively [10,14]. However, despite the decrease in varicella incidence among vaccinated children, there has been an increase in herpes zoster occurrence [15]. The live-attenuated herpes zoster vaccine is effective in preventing the development of herpes zoster and its complications, including post-herpetic neuralgia [16]. The shingles prevention study by Schmader et al. demonstrated that the vaccine reduced the incidence of shingles by 51% and post-herpetic neuralgia by 67% in individuals aged 60 years and older [16]. However, its efficacy may decline beyond five to eight years post-vaccination, as reported by Morrison et al. [17]. Therefore, administering the zoster vaccine to older adults is crucial to protect against herpes zoster and its debilitating complications [17].

Many physicians now face life-threatening varicella cases with diagnostic uncertainty. Our study aims to establish an early clinical diagnosis of myocarditis in VZV infection to decrease the associated morbidity and mortality. In this review, we will focus on the early diagnosis of myocarditis caused by the VZV.

## Review

Methodology

Search Strategy and Selection Criteria

This systematic review was conducted to evaluate the relationship between varicella viral infection cases and the occurrence of viral myocarditis. The literature review was performed by searching different databases, including PubMed and Google Scholar, for articles published between 1982 and 2022 but without language constraints using the search terms “Varicella” OR “Chicken Pox” AND “Myocarditis” AND “Varicella Complications” AND “Varicella vaccine.”

Inclusion and Exclusion Criteria

Our systematic review focused on case reports and observational studies involving individuals who were diagnosed with both varicella viral infection and viral myocarditis. Our aim was to examine the early clinical diagnosis of myocarditis in varicella-infected patients across all age groups, from infants as young as two days old to the elderly. We excluded studies that did not report a relationship between varicella infection and myocarditis from our analysis.

Data Extraction

Standardized data extraction forms were used to collect information on age, clinical presentation, symptom duration, and vaccination status. The authors’ findings on the association between varicella and myocarditis were analyzed qualitatively.

Data Analysis

The data analysis was stratified according to several factors, including the age group of the participants, the time interval between the onset of VZV symptoms and clinical presentation, and the duration between VZV infection and the development of viral myocarditis. Furthermore, the analysis also considered the immune status of the participants, particularly regarding whether immunization with the varicella vaccine had any effect in preventing the development of myocarditis.

This study focuses on the rare yet severe complication of myocarditis that can arise from varicella infection. To investigate this, we reviewed case reports and observational studies that featured individuals with varicella infection and myocarditis, with the goal of including all such cases in our analysis.

**Figure 1:**
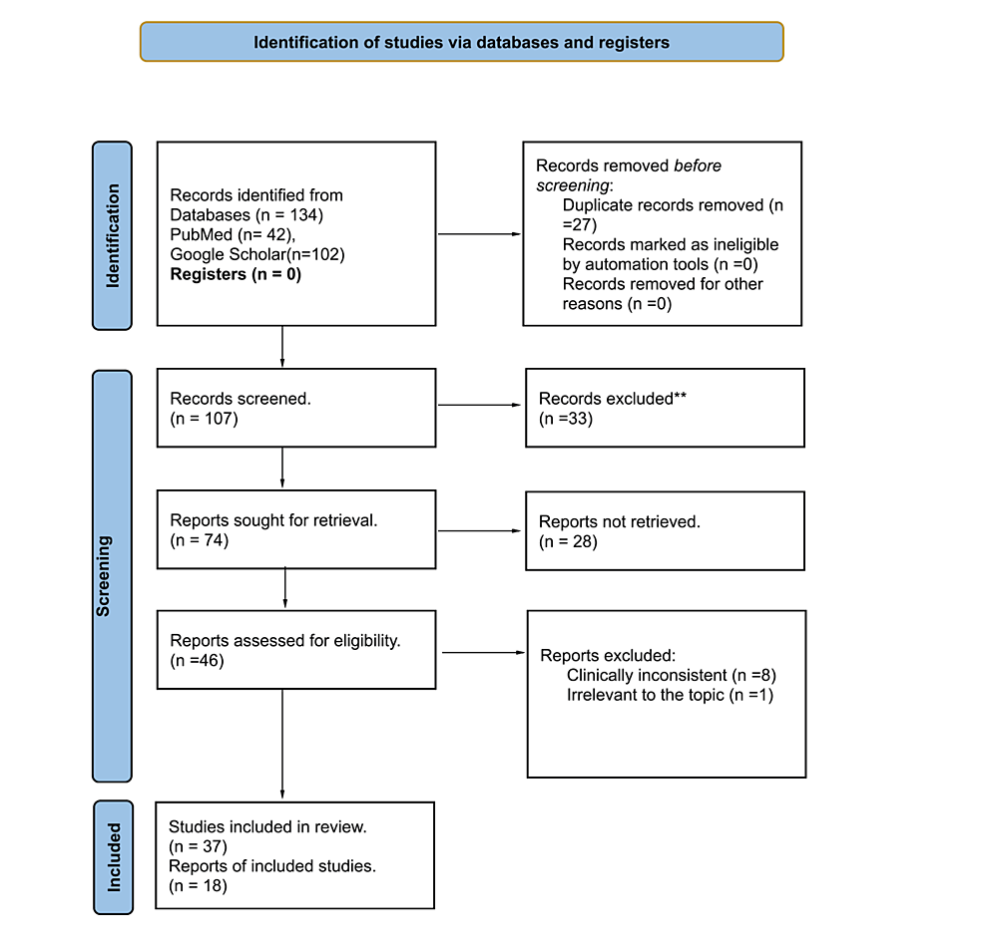
Flow of information through the different phases of the systematic review.

Results

We identified a total of five studies and 13 cases related to varicella myocarditis. Our analysis revealed the below findings.

One case of combined ventricular fibrillation (VF) and preceding VZV myocarditis was seen in a 46-year-old male patient. He presented with recurrent episodes of collapse and a VF arrest requiring emergency cardioversion by the emergency room (ER) team. He was treated with intravenous amiodarone for 24 hours and intravenous acyclovir for 10 days. An uneventful recovery followed this. The patient presented eight years later with a syncopal event lasting less than a minute. His resting electrocardiogram (EKG) showed sinus rhythm with a regular QTc interval. The cardiac MRI revealed mid-wall myocardial enhancement at the basal inferior and inferolateral walls consistent with myocarditis. An automatic implantable cardioverter defibrillator was inserted. Two years later, he had VF episodes, and interrogation of the device showed two shocks for VF. This further shows how one of the severe complications of VZV can affect cardiac function in the long run [18]. Another case is of a 27-year-old man who presented with fever, chest pain, and vesicular-pustular eruptions on his face and trunk and presented to the ER with tachypnea. The patient was started on parenteral acyclovir and steroids for seven days. He was discharged on oral acyclovir for seven days with an uneventful follow-up period of six months. The follow-up EKG at the time of discharge compared to the EKG after three months showed near-complete resolution of ST-T changes, and the two-dimensional echocardiogram at three months showed improvement in left ventricular function from 40% to 53% [19].

Early diagnosis and adequate identification of potential upcoming complications remain essential concepts that should be worked on tirelessly. This was seen in two cases. The first was of a 23-year-old male who was admitted with chest pain. The EKG showed ST elevation in leads DI, aVL, and V2-V6. Troponin-T was 1.1 ng/mL, and the left ventricular systolic function was globally reduced (ejection fraction of 45%). The patient was treated with thrombolytics. Twenty-four hours after admission, pruritic vesicles appeared with explicit content and were surrounded by a pink halo on his face, head, and torso, suggesting VZV infection [20]. The second case was of a 23-year-old male who developed clinical symptoms of myopericarditis a week after diagnosis of VZV. The rash was localized in the left upper part of the thorax. The EKG mimicked acute myocardial infarction accompanied by retrosternal chest pain and fever. A dynamic pattern of troponin-I was released, and slow normalization of EKG was observed. Serial echocardiography showed normal left ventricular function, transient changes in the echogenicity of the interventricular septum, and small pericardial effusion. On MRI, subepicardial and intramyocardial areas of late gadolinium enhancement were found. The patient was treated with intravenous acyclovir. No late sequels of the disease were observed [21]. This is an excellent example of early detection and mitigation of late complications. An interesting study done in 1983 showed that 31 immunocompromised patients had varicella infection, and although 15 patients had a severe visceral infection, out of those 15, only one developed myocarditis. In patients with severe involvement, intense abdominal pain was the first sign of dissemination and the warning sign of an upcoming rare complication involving the heart and eventually leading to myocarditis [22].

In 1988, Waagner and Murphy identified 18 cases of VZV myocarditis in immunocompetent children (ages 10 months to 14 years) over thirty-three years of age. The majority presented within three to five days from the onset of the typical vesicular exanthem. Of the 18 children with VZV myocarditis, seven had a fatal outcome. Four of the seven children presented with cardiac arrest or succumbed to severe myocardial dysfunction within 24 hours. The remaining three patients died of non-cardiac etiologies. The age range of the remaining six children was 11 months to 14 years, and all cases involved immunocompetent children [23]. On autopsy, a six-year-old girl had disseminated VZV infection, including myocardial involvement [24]. Two additional children, ages 11 months and four years, had evidence of ectopic junctional tachycardia and transient heart block, respectively [24,25]. An infant died as a direct result of the arrhythmia [26]. Another case of a two-year-old girl who was airlifted but dead-on-arrival was unwell for four days before her death. After her initial prodromal illness, the characteristic exanthema of VZV infection developed. She was managed at home with antipyretics and oral hydration. On day four of the illness, the girl presented with drowsiness, irritability, and tachypnea. A cardiac arrest occurred in the office, and despite resuscitation efforts, she died before arrival at the hospital. One month before her death, she was admitted with a common respiratory tract infection, but the chest radiograph was unremarkable, and she had an average heart size. However, the postmortem finding included a massively dilated and hypertrophied heart [27].

Previous vaccination also affects the diagnostic modality of the disease. One good example is a previously vaccinated 15-year-old boy who was admitted to the emergency department for chest pain, tachycardia, and hypotension after exposure to a child with chickenpox one week prior. An EKG showed sinus tachycardia. Cardiac biomarkers were elevated, and echocardiography revealed left ventricular apical, inferolateral, septal hypokinesis, and mitral regurgitation. The VZV serum IgM antibody was positive. Four-week follow-up showed significantly elevated IgM and IgG VZV antibodies, which helped in the diagnosis [9]. Few studies showed that after VZV infection, IgG and IgM antibodies appear two to five days after the rash and show the highest titers at two to three weeks. The VZV IgM antibody levels then rapidly decrease and cannot be detected one year after infection. The IgG antibody levels gradually decrease, showing positive test results for several years [28].

One case of a 17-year-old boy who showed atypical EKG findings of pericarditis and remarkably elevated cardiac biomarker levels: peak cardiac troponin I, 37.2 ng/mL, total creatine kinase, 1,209 U/L, and creatine kinase-MB fraction, 133.6 ng/mL. After the results of coronary angiography reliably excluded ischemia and myocardial infarction, the patient was diagnosed with varicella myopericarditis [5]. Similar episodes were also seen in other studies, such as in a 16-year-old male who presented with symptoms and investigations suggestive of acute myocardial infarction. The patient had been infected with VZV five days before presentation, and varicella myocarditis was suspected. This was confirmed following treatment and positive varicella serology [29]. Another case involved a 12-year-old male child with Down syndrome who recovered from congenital heart disease and later developed severe varicella myocarditis. His clinical presentation at admission mimicked acute coronary syndrome. Analysis of this case provides insights into several aspects of varicella myocarditis [30].

Discussion

Viral myocarditis is an increasingly understood cause of heart failure [14]. VZV, the virus responsible for chickenpox infection, is usually self-limiting. In rare cases, particularly in immunocompromised individuals following heart transplants, VZV infection can cause myocarditis, arrhythmias, and pericardial effusion [31]. The VZV mortality rate is approximately 25-fold higher among adults than children one to four years of age [32]. The risk of both severe complications and mortality is increased among infants, adults, and immunocompromised individuals of any age [27].

Early prognosis and suitable remedy for this uncommon type of myocarditis can lead to early recovery. In addition, early identification of VZV may prevent the upcoming sequel [33]. One question remains significantly important: how do we prevent this from occurring repeatedly? MRI holds promise as an effective non-invasive diagnostic tool. There is a lack of controlled clinical trials for both acute pericarditis and myopericarditis. On follow-up, most of these cases had accurate normalization of echocardiography, EKG, laboratory testing, and functional status, although up to 14% may report atypical, non-limiting chest discomfort. Unfortunately, few data have been published on myopericarditis [34].

Additionally, the diagnosis of VZV myocarditis in children is not straightforward. Three parameters play a role in the disease progression, namely, the patient’s age, vaccination status, and immune competency [7]. However, the development of tachycardia and chest pain in patients with varicella should signal the possibility of myocarditis. The clinical findings of varicella infection, especially in adolescents, may not be present at the time of admission. In atypical clinical situations, myocardial infarction and acute coronary syndrome should be excluded before proceeding with treatment [9]. Numerous complications are reported to cause mortality in childhood varicella infection. Approximately half of all fatalities are secondary to pneumonia and central nervous system complications. Deaths from cardiac complications are likely underreported. A cardiac evaluation should be considered for all children with clinical VZV infection who are significantly unwell considering that patients may present up to two weeks after the onset of the vesicular exanthem [27].

The wide range of severity and variety of cardiac involvement in varicella leads us to the assumption that subclinical forms of myocarditis are probable during epidemics of varicella, similar to those described and associated with other infections [35]. The treatment of varicella infection depends on the patient’s age, immune status, and severity of symptoms. Symptom relief is the main focus of treatment, which can be achieved using analgesics, antipyretics, and topical agents to mitigate skin discomfort [36]. Antiviral drugs, such as acyclovir, valacyclovir, or famciclovir, may be prescribed in severe cases or when complications arise and can be beneficial in reducing the illness duration and preventing further complications [31]. The recommended dose of acyclovir for the treatment of varicella in immunocompetent individuals is 800 mg orally, five times daily for seven to ten days [37]. Intravenous acyclovir therapy may be necessary in severe or complicated cases, with a recommended dose of 5-10 mg/kg every eight hours for seven to ten days. The route and frequency of acyclovir therapy may be adjusted based on the patient’s age, weight, and renal function. It is crucial to initiate antiviral therapy within 24-72 hours of symptom onset for optimal effectiveness [31]. Less common but more serious side effects include seizures, hallucinations, and kidney damage. Monitoring the patient’s cardiac function during and after treatment is also recommended [31]. Immunocompromised individuals may require more aggressive management strategies, including hospitalization, intravenous antiviral therapy, and close monitoring for potential complications [31]. However, there is no evidence to support the effectiveness of acyclovir due to the rarity of VZV myocarditis [21,27].

Limitations

During our comprehensive review of varicella infection and myocarditis, we encountered a limitation regarding the number of studies available on the effectiveness of the varicella vaccine in reducing the risk of subsequent myocarditis. While our study sheds light on the rare but serious complication of myocarditis associated with varicella infection, further research is required to determine the effectiveness of the varicella vaccine in preventing this complication. Given the urgency of this issue, there is a need for more studies on VZV cases, uncommon yet serious sequelae, and the efficacy of the varicella vaccine.

## Conclusions

In summary, this study aimed to identify the correlation between early clinical diagnosis of myocarditis in VZV-infected patients for better outcomes and reduction of associated mortality. This study included case reports and observational studies about this association based on our selection criteria. Many of our findings indicated that a positive relationship between earlier diagnoses and treatment of myocarditis in VZV-infected patients can be linked to more significant outcomes and fewer fatalities. Viral myocarditis is one of the leading causes of heart failure, with VZV being responsible for myocarditis in both immunocompetent and immunocompromised patients. It was found that the VZV mortality rate is higher among adults, infants, and immunocompromised patients.

Most patients presented with symptoms such as tachycardia, tachypnea, chest pain, and arrhythmia, along with EKG changes and elevated biochemical markers. In rare cases, vesicular skin manifestations may develop after cardiac symptoms in infected patients. The timeline for the clinical presentation of myocarditis clinical presentation ranges from immediately to one week. In some cases, it may present as late as eight years following the VZV infection.

We suggest that every patient with varicella infection should have a cardiac examination, including an EKG, during the acute phase of the disease.

Although immunization against varicella infection played a role in minimizing the severity of the disease, there was no data to support the impact of the varicella vaccine in preventing myocarditis. To better understand this correlation, a more extensive cohort study is much needed on this topic.
